# Supplementary figures and images for: An atlas of Caenorhabditis elegans chemoreceptor expression
Source: PLoS Biol. 2018 Jan 2;16(1):e2004218. doi: 10.1371/journal.pbio.2004218 (PMC5749674; doi:10.1371/journal.pbio.2004218)

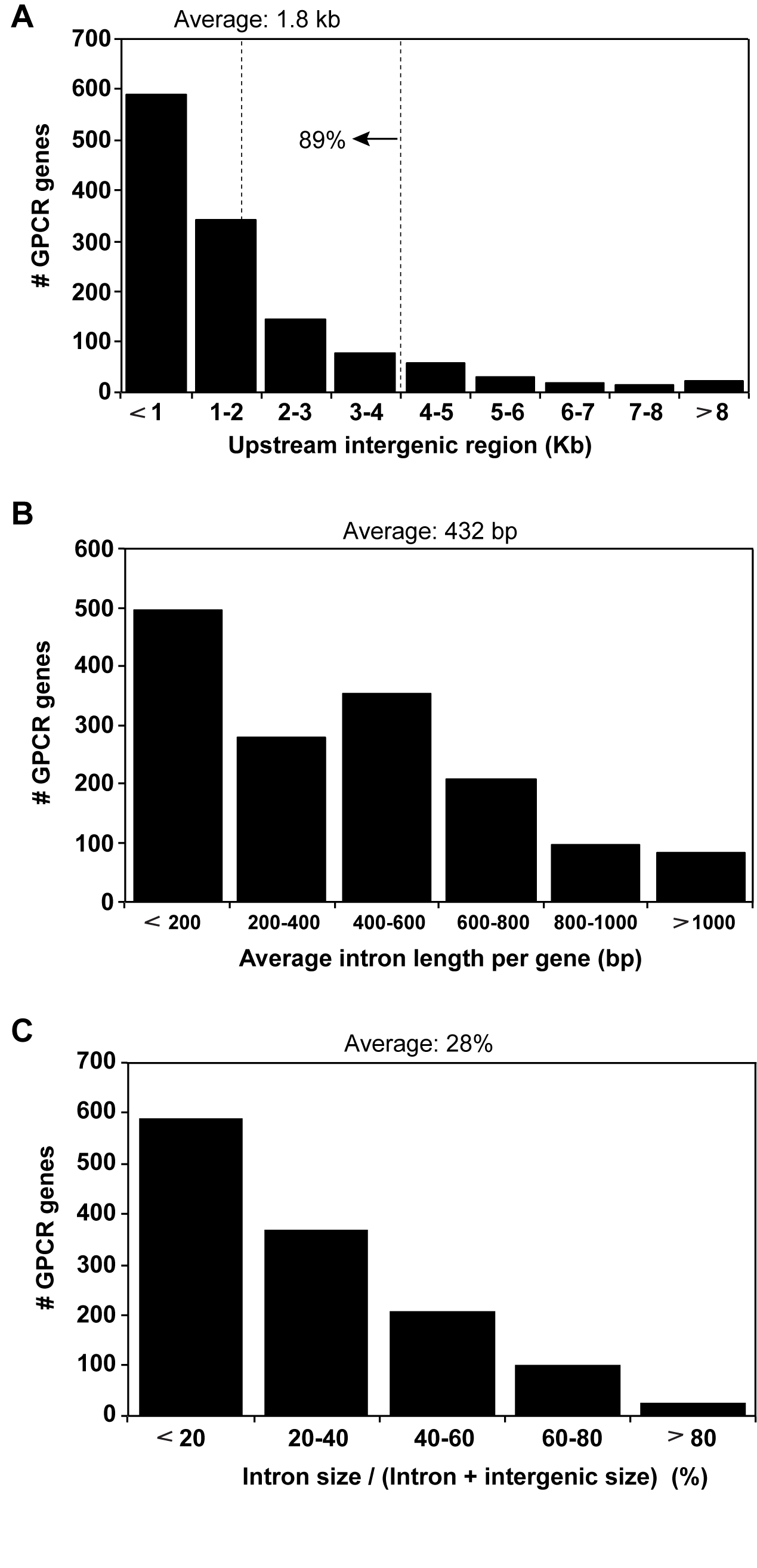

Supplement: S1 Fig — (A) Histogram of upstream intergenic region distances of all C. elegans csGPCR genes. The average size of the 5’ intergenic region (= distance to next gene) is 1.8 kb. Eighty-nine percent of all loci have a 5’ intergenic region smaller than 4 kb. (B) Histogram of average combined intron length (bp) per GPCR gene. (C) The intergenic region of the majority of GPCR is substantially larger than the combined intronic region. bp, base pair; csGPCR, chemosensory-type GPCRs; GPCR, G-protein-coupled receptor. (TIF) [file pbio.2004218.s001.tif]
